# Perspectives of community-dwelling older adults with chronic diseases on Baduanjin practice: A qualitative study

**DOI:** 10.1371/journal.pone.0351557

**Published:** 2026-06-12

**Authors:** Haixu Ji, Wei Li, Jinhua Zhang, Xuyan Liu, Jing Wang, Guanglei Dong

**Affiliations:** 1 School of nursing, North Henan Medical University, Xinxiang, Henan, China; 2 Department of nursing, Henan Medical University, Xinxiang, Henan, ‌‌China; 3 Library, North Henan Medical University, Xinxiang, Henan, ‌‌China; Mae Fah Luang University School of Anti Aging and Regenerative Medicine, THAILAND

## Abstract

**Background:**

With the accelerating aging of China’s population, Baduanjin has been promoted as a community-based exercise to enhance public health, particularly among older adults with chronic diseases. As a traditional Chinese exercise with a long history and profound cultural connotations, Baduanjin has attracted a large number of practitioners. However, the factors underlying the sustained practice of Baduanjin remain insufficiently explored.

**Objective:**

This study aims to explore the factors underlying the persistence of older adults with chronic diseases in practicing Baduanjin.

**Methods:**

A qualitative research approach was adopted in this study. 25 practitioners participated in semi‑structured face‑to‑face interviews. Thematic analysis was employed to analyze the data and generate core themes.

**Results:**

The factors driving community-dwelling older adults with chronic diseases to persist in practicing Baduanjin were analyzed across five dimensions: perceived safety and learning-practice ease, improvements in physical health, promotion of mental well-being, enhancement of social functioning, and appreciation of traditional culture.

**Conclusion:**

Community-dwelling older adults with chronic diseases maintain long-term Baduanjin practice not only due to its perceived safety and ease of learning and practice, but also because it embodies the essence of traditional Chinese culture. Moreover, regular practice contributes to improved physical health, promoted mental well-being, and enhanced social functioning in this population. Accordingly, Baduanjin shows considerable potential as a community-based exercise intervention to support health promotion among community-dwelling older adults with chronic diseases.

## Introduction

In China, the prevalence of chronic diseases among older adults has reached 81.1%, accounting for approximately 179.9 million individuals [1]. This demographic is not only burdened by persistent physical impairments but also faces a higher risk of somatic symptom disorder, along with comorbid depression and anxiety disorders [2]. Consequently, the physical and mental health challenges confronting older adults with chronic diseases (OAwCD) have become increasingly prominent, which has further led to a substantial surge in demand for healthcare services. Notably, healthcare expenditures among the population aged 65 years and above are the highest across all age groups [3]. Given that OAwCD often experience multiple impairments in physical function, psychological state, emotional regulation, and cognitive ability, there is an urgent need for accessible, affordable, timely, and high-quality healthcare interventions to address their complex needs [4].

Accumulating evidence has confirmed that exercise-based therapies and rehabilitation programs play a crucial role in chronic disease management, as they can effectively reduce hospitalization rates, improve quality of life, and enhance cost-effectiveness [5–7]. As a key lifestyle factor, physical exercise not only influences the pathogenesis of various chronic diseases, including cardiovascular, metabolic, oncological, and neurological conditions but also has the potential to modify their developmental trajectory [8–9]. However, non-adherence to exercise regimens is prevalent in the management of nearly all chronic diseases [10]. Coupled with the lack of effective external supervision and systematic management [11], it remains challenging for OAwCD to maintain regular physical exercise, which hinders the achievement of optimal rehabilitation outcomes.

Furthermore, the treatment and rehabilitation of chronic diseases represent long-term, continuous processes that require sustained intervention [12]. Driven by shrinking family sizes, changing living arrangements, and particularly the out-migration of adult children, the role of families in elderly care has gradually diminished. In response to this trend, China has been actively constructing institutional and community-based nursing infrastructure, which serves as both a substitute for and a complement to traditional family care [13].

Among various community nursing interventions, community-based exercise is widely recognized as one of the most cost-effective strategies, as it can assist in managing cancer-related fatigue [14], improve quality of life (QOL) in adult cancer survivors [15], enhance motor function in patients with Parkinson’s disease [16], and facilitate improved self-management of the disease [17]; however, despite these well-documented benefits, existing community-based exercise programs have paid minimal attention to the investigation of qualitative outcomes [18], which limits a comprehensive understanding of their holistic effectiveness and acceptability among participants.

Baduanjin (Eight-Section Brocade, ESB), a traditional Chinese mind-body aerobic exercise originating in the Song Dynasty, integrates the theoretical system of Traditional Chinese Medicine (TCM), health-preserving concepts, and traditional martial arts movements [19]. As an ancient cultural heritage with a millennia-long transmission history, Baduanjin embodies the Chinese nation’s pursuit of physical health and harmonious coexistence between the human body and nature [20]. Characterized by its simplicity, moderate intensity, and low physical burden, it is applicable to individuals across all age groups, with particular suitability for older adults and populations with chronic diseases [21–22].

A growing body of studies has demonstrated the multifaceted health benefits of Baduanjin. For instance, it has been shown to improve balance, leg strength, and mobility, serving as a safe and sustainable home-based exercise for individuals with chronic stroke [21]. A recent systematic review reported that among various mind-body exercises, while most can improve blood glucose and lipid profiles, only Tai Chi and Baduanjin significantly increase high-density lipoprotein cholesterol levels in patients with type 2 diabetes mellitus (T2DM) [23]. Additionally, Baduanjin has been proven to enhance bone mineral density in postmenopausal women with osteoporosis [24], improve exercise endurance in patients with chronic obstructive pulmonary disease (COPD) [25], reduce blood pressure in hypertensive patients [26], restore intestinal flora balance in prediabetic individuals [27], alleviate cancer-related fatigue [28], improve sleep quality in older adults [29], and relieve neck, back, and knee pain [30–32].

Beyond physical health benefits, Baduanjin also exerts positive effects on mental health. Evidence suggests that it can effectively ameliorate depressive symptoms by regulating the dysregulated expression of long non-coding RNAs (lncRNAs), messenger RNAs (mRNAs), and circular RNAs (circRNAs) [33]. Other studies have reported that Baduanjin can reduce hemoglobin A1c levels and alleviate symptoms of anxiety, depression, and hostility in patients with T2DM [34–35], as well as significantly relieve anxiety and depression in post-operative breast cancer patients [36]. Owing to these comprehensive health advantages, Baduanjin has become a preferred form of exercise among many OAwCD.

Despite the proven benefits of Baduanjin and its high acceptance among OAwCD, the factors influencing long-term adherence to Baduanjin practice remain inadequately explored. Long-term adherence is the key to ensuring the sustained health effects of exercise interventions, and clarifying the determinants of adherence is essential for developing targeted strategies to promote the effective use of Baduanjin in chronic disease management among older adults. Therefore, this study aims to explore the determinants underlying the sustained practice of Baduanjin among community-dwelling OAwCD through semi-structured in-depth interviews. The findings are expected to elucidate the lived experiences and intrinsic motivational factors of this population in engaging in Baduanjin, enrich the phenomenological understanding of traditional Chinese health-enhancing exercises, and provide empirical evidence for the development of targeted interventions to facilitate healthy aging in OAwCD.

## Methods

### Study design

This study adopted a qualitative descriptive design and employed Braun and Clarke’s six-phase thematic analysis to analyse the interview data [37]. It was selected because it is uniquely suited to capturing lived experiences and intrinsic motivational processes. Furthermore, it offers epistemological flexibility while upholding methodological rigor, making it particularly appropriate for phenomenologically oriented inquiry into subjective health behaviors [38–39]. As a robust qualitative analytic approach, TA enables the systematic identification, interpretation, and reporting of patterned meanings across datasets, thereby supporting in-depth exploration of why and how older adults maintain long-term engagement in Baduanjin practice.

### Sampling and recruitment

Participants were recruited from two communities in Xinxiang between July and August 2024; with the assistance of community managers as intermediaries, potential participants were approached and invited face‑to‑face during routine Baduanjin practice sessions. Prior to data collection, rapport and trust were established with respondents, and written informed consent was obtained from all eligible participants after a full explanation of the study purpose, procedures, and ethical considerations. To ensure comprehensive and reliable data, maximum variation sampling combined with purposive sampling was adopted to enroll practitioners with diverse demographic characteristics, including age, gender, and educational level.

Inclusion criteria for Baduanjin practitioners were as follows: (1) aged 65 years or older; (2) having engaged in continuous Baduanjin practice for at least 6 months (to ensure sufficient practical experience for meaningful contribution); (3) diagnosed with chronic non-communicable diseases.

Data saturation, as defined by Moser and Korstjens (2018), refers to the point at which qualitative data collection can be concluded because no new analytical information emerges from additional data, and the research phenomenon has been comprehensively elucidated with maximum informational coverage [40]. In the present study, data collection reached this saturation criterion when no new thematic categories were identified following the interview with the 22nd Baduanjin practitioner. To rigorously confirm the attainment of data saturation, three further eligible Baduanjin practitioners were subsequently interviewed, with no novel analytical insights generated from these additional interviews. Finally, 25 Baduanjin practitioners were enrolled in this study and assigned sequential codes from P1 to P25 for the ensuing qualitative analysis. The demographic characteristics of the participants are presented in Table 1.

**Table 1:** Demographic characteristics of the practitioners of Baduanjin (n = 25).

No	Age	Gender	Education	Average number of exercises per day	Practice duration (months)	Chronic diseases
P1	70	male	Junior high school	3	18	Hypertension; hypothyroidism
P2	72	Female	Junior college	2	17	Hypertension; Hypercholesterolemia
P3	67	Male	junior college	3	13	Hypertension; Cerebral infarction
P4	69	Female	Junior high school	2	24	Coronary Heart Disease; Osteoporosis
P5	68	Male	Junior college	3	11	Hypertension
P6	67	Female	Junior high school	2	14	Breast Cancer
P7	66	Female	Senior high school	1	7	Impaired glucose tolerance; Cyst of Liver
P8	71	Female	Junior high school	2	10	Hypertension; T2DM; Coronary Heart Disease
P9	74	Male	Senior high school	2	12	Retrogression gonarthritis
P10	69	Male	Primary school	2	24	Hypertension; T2DM
P11	77	Female	Junior high school	1	10	COPD
P12	73	Female	Primary school	2	12	Gastric cancer
P13	68	Female	Junior high school	2	24	Hypertension; T2DM
P14	75	Female	Undergraduate college	1	12	Cervical spondylosis; Degenerative osteoarthritis
P15	71	Female	Primary school	2	24	Arthritis; Osteoporosis
P16	68	Female	Senior high school	2	14	Coronary Heart Disease
P17	67	Male	Senior high school	2	8	Impaired glucose tolerance; Benign Prostatic Hyperplasia
P18	68	Female	Senior high school	2	13	Osteoporosis; Anemic
P19	65	Female	Junior high school	2	60	Hyperlipidaemia
P20	74	Female	Primary school	2	9	Gastritis; Hypertension
P21	67	Male	Undergraduate college	1	36	Hypertension
P22	66	Female	Senior high school	2	12	T2DM
P23	72	Female	Junior high school	2	24	Hypertension
P24	71	Female	Senior high school	1	12	Hypertension; T2DM
P25	65	Female	Undergraduate college	2	72	Hyperlipidaemia

### Data collection

Data were collected via face-to-face, semi-structured in-depth interviews. With the interviewees’ informed consent, the entire interview process was audio-recorded. During interviews, researchers made flexible adjustments to the sequence and phrasing of questions based on the interview guide and the interviewees’ actual responses, while appropriately following up on valuable insights. All interactions adhered to the principle of non-directionality, ensuring that interviewees’ expressions were respected without leading or implied suggestions. Interviews were conducted in the community setting where Baduanjin is regularly practiced, with each session lasting 30–40 minutes. The interview guide used in this study was developed based on researchers’ long-term observational data and refined iteratively according to feedback from three pre-interview sessions with Baduanjin practitioners. The final version of the interview guide is provided in Supplementary File 1.

### Data analysis

Interview recordings were transcribed and compiled into textual data within 24 hours post-interview, with repeated review of the original recordings to verify transcript accuracy. Two researchers (Haixu and Jinhua) independently conducted data analysis following Braun and Clarke’s thematic analysis [37]. They repeatedly read the transcripts to immerse themselves in the data, extracted meaningful statements, and performed initial coding. All codes and potential themes were then cross-compared. Any discrepancies in coding and theme interpretation were resolved through in-depth discussion until a consensus was achieved.

To ensure the identified themes authentically reflected participants’ lived experiences, systematic member checking and triangulation validation were adopted. After initial theme extraction, three researchers (Haixu, Jinhua, and Wei) reviewed and compared the analytical results, resolved interpretive discrepancies through in-depth discussion, and reached a consensus on the preliminary themes. Ambiguous contents in the interview recordings were further clarified via follow-up face-to-face communication with participants to confirm their original intentions. All participants were subsequently provided with the final interview transcripts and primary and sub-themes in Chinese (consistent with the interview language) for formal member checking. Participants were invited to verify the accuracy of the transcripts and the consistency of the themes; their feedback was collected and discussed by the research team, and minor revisions were made accordingly to ensure the findings faithfully represented participants’ genuine perspectives.

Since the interviews and data analysis were conducted in Chinese, the study quotes were first translated into English by two researchers (Haixu and Jinhua). To ensure translation accuracy and avoid bias, the translated content was cross-checked and back-translated into Chinese by another two researchers (Wei and Xuyan). The back-translated version was then compared with the original transcripts to ensure consistency and eliminate errors.

### Ethical considerations

This study was approved by the Academic Ethics Committee of North Henan Medical University (formerly Sanquan College of Xinxiang Medical University), Approval No. 2024061501. All procedures performed in this study adhered strictly to the ethical guidelines outlined in the Declaration of Helsinki. Prior to the commencement of interviews, participants were provided with a comprehensive explanation of the study’s purpose, content, and confidentiality protocols. Written informed consent was obtained from each participant prior to their involvement. To safeguard the privacy of participants, all identifying information, including their real names, was anonymized using numerical codes.

## Results

The interview findings revealed five core themes regarding the factors that contribute to the persistence in Baduanjin practice, namely: perceived safety and learning-practice ease, improvements in physical health, promotion of mental well-being, enhancement of social functioning, and appreciation of traditional culture. The primary themes and their corresponding sub-themes derived from the interviews are presented in Table 2.

**Table 2:** The main themes and sub-themes of the interview.

Main Themes	Sub-themes
Perceived safety and learning-practice ease	Perceived safety
	Ease of learning and practice
Improvements in physical health	Increased range of motion
	Enhanced balance and coordination
	Alleviation of somatic symptoms
Promotion of mental well-being	Improved mood state
	Maintenance of cognitive function
	Reduced negative psychological states
Enhancement of social functioning	Increased sharing and communication
	Facilitation of conflict resolution
	Reduction of social isolation and loneliness
Appreciation of traditional culture	Recognition of traditional health-preserving culture
	Connection with Traditional Chinese Medicine culture

### Theme I perceived safety and learning-practice ease

#### Perceived Safety.

Baduanjin, a traditional Chinese health-preserving exercise, is primarily aimed at tendon conditioning, bone strengthening, qi nourishing, and physical fitness enhancement. Characterized by non-complex technical movements, it avoids intense running or jumping that may induce safety incidents, thereby exhibiting favorable safety profiles both during and post-practice. The movements of Baduanjin are performed in a slow and gentle manner, with an emphasis on bodily integrity and coordination. This movement pattern exerts minimal mechanical stress on joints and muscles, significantly reducing the risk of sports-related injuries. Furthermore, the range of motion (ROM) and exercise intensity can be flexibly adjusted based on individual physical status, which serves as an effective preventive measure against muscle strains or joint sprains caused by excessive exertion.

*“I am very interested in the five-animal exercise. However, considering its numerous sections and complex movements, I was worried that I couldn’t learn it well and was also afraid of falling. In contrast, Baduanjin only has eight movements. I’m not worried about not being able to learn it, nor am I afraid of suddenly falling while practicing. Moreover, its exercise effects are quite remarkable. I feel that Baduanjin is especially suitable for me.” (P2).*

*“Practicing Baduanjin does not damage the joints. Previously, I suffered from knee pain due to square dancing and underwent a right knee joint replacement surgery. Since then, I have been practicing Baduanjin to promote recovery and maintain good health.” (P9).*

#### Ease of learning and practice.

Baduanjin exhibits extremely low demands on practice venues and can be performed in nearly all types of environments, including both indoor spaces and outdoor settings, provided that there is sufficient space to allow for full body stretching. Notably, it does not require any specialized equipment; practitioners only need to wear comfortable, loose-fitting clothing and sports shoes to engage in the exercise at any time and in any location. In terms of motor characteristics, Baduanjin movements are straightforward, and its practice rhythm is relatively slow—features that align well with the physical movement patterns and cognitive capabilities of the elderly population.

*“Baduanjin is not restricted by weather or venue. On sunny days, we prefer to practice in the community square, which has lush greenery and fresh air, allowing us to get close to nature and unwind. When the weather turns bad, we go to the community senior center or practice at home.” (P5).*

*“Baduanjin is easy to learn and can be practiced at any time” (P9).*

*“The practice rhythm of Baduanjin is relatively slow, and the names of its eight sections are highly general and instructive. Thus, during practice, there is sufficient time to understand and execute each movement.” (P17).*

### Theme II Improvements in physical health

#### Increased range of motion.

The movements of Baduanjin are characterized by elegant continuity and sophisticated structural design, enabling comprehensive engagement of the head, neck, trunk, limbs, joints, muscles, and ligaments. This holistic activation and stretching of all bodily segments effectively mitigates age-related deterioration of physical function and the onset of motor dysfunction.

*“Last year, I suffered a cerebral infarction. At that time, I couldn’t straighten my right fingers, my right leg was weak, and I walked crookedly, unsteadily, and with poor coordination. After more than a year of practice, my fingers can now straighten fully, and their bending and straightening functions have basically returned to the pre-illness state, and my right leg is strong and moves flexibly when I walk.” (P3).*

*“I am a patient suffering from cervical spondylosis and shoulder periarthritis. The doctor suggested that I practice Baduanjin. After practicing for one year, the pain in my neck and shoulders has been relieved, and I can raise my arms over my head.” (P14).*

#### Enhanced balance and coordination.

Long-term practice of Baduanjin may enhance muscle strength, expand the range of joint motion, and improve balance and coordination abilities.

*“I used to feel weak and was afraid of falling while being active. After practicing Baduanjin with others, I found that my body became stronger and more flexible. I could maintain balance more easily and was less afraid of falling.” (P13).*

#### Alleviation of somatic symptoms.

Each movement of Baduanjin is scientifically designed to target the functions of specific zang-fu organs (viscera). Coupled with the requirement of coordinating with deep, slow, uniform, and tranquil breathing, it can alleviate somatic symptoms in OAwCD by enhancing visceral functions.

*“I had high blood lipids and used to suffer from constipation. Later, I adjusted my diet and practiced Baduanjin twice a day, in the morning and afternoon. Now I can defecate regularly once a day.” (P2).*

*“After undergoing radical surgery for breast cancer, I was in a very depressed state of mind. I didn’t feel like talking to people, had no appetite, and my weight was steadily dropping. [...]. I have started to come to the square every morning to practice Baduanjin with other practitioners. I feel much better now, and my appetite has returned.” (P6)*

*“I was diagnosed with COPD a year ago. I was forced to quit smoking, and at first, I felt very bored. When I saw other people practicing Baduanjin in the community square, I joined them. Unexpectedly, I found joy again. Now, I can breathe a lot easier.” (P11).*

*“I am a little anemic. I heard that Baduanjin can unblock the meridians and promote qi and blood circulation, so I joined others in practicing it. After each practice session, I break into a slight sweat, and my whole body feels warm. I also feel particularly refreshed, and my hands and feet are warmer than before.” (P18)*

### Theme III Promotion of mental well-being

#### Improved mood state.

In the practice of Baduanjin, practitioners are advised to maintain a tranquil mental state, with focused attention on the ongoing movements and breathing patterns, thereby eliminating extraneous distractions. This state of heightened mind-body integration not only facilitates the alleviation of psychological stress but also promotes emotional calmness and enhances mood stability.

*“We opted to practice Baduanjin as the sun was rising, accompanied by soothing music. After the practice, I felt refreshed and delighted.” (P10).*

*“One morning, my husband and I quarreled, and I was extremely angry. Subsequently, I went to practice the Baduanjin. While practicing, I was instructed to relax my entire body and breathe evenly. I made an effort to do so. Gradually, my breathing slowed down and eventually became calm and natural. During the practice, I seemed to have forgotten the anger I had just experienced, and I felt less angry after finishing the practice.” (P19).*

*“I engage in self-practice of Baduanjin, typically selecting a quiet and aesthetically pleasant garden setting. This environment facilitates enhanced concentration on both movement execution and respiratory regulation. Such practice not only induces a state of inner tranquility but also fosters a perceived sense of harmony with the natural surroundings.” (P25).*

#### Maintenance of cognitive function.

Baduanjin is firmly grounded in TCM theory. To further optimize the exercise efficacy of Baduanjin, some older adults proactively acquire relevant theoretical knowledge of TCM. This behavior not only enables them to more accurately grasp the essential movement requirements and the core principles of health preservation but also, more importantly, helps maintain their cognitive learning ability to a certain extent. Concurrently, Baduanjin practice can promote the maintenance of clear thinking, sound memory, and robust concentration in the elderly, thereby contributing to the preservation of cognitive function.

*“I went to the University for the Aged to learn about TCM. I studied the Meridian Theory and Zang-Fu Theory. This knowledge can be applied in practice and shared with others, which makes me feel very fulfilled and happy.” (P17).*

*“When practicing Baduanjin, first and foremost, I must keep in mind the concise instructions for each of the eight sections. Each section should be practiced six times, except for the last section, which requires shaking the foot seven times. The left-right symmetry movement involves three repetitions on the left side and three repetitions on the right side. After practicing each section, practitioners need to return to the starting position. During the practice, I must focus intently; otherwise, I will lose track of the number of repetitions. I believe that by practicing in this way, I can maintain my concentration, comprehension, and memory as I age.” (P24).*

#### Reduced negative psychological states.

The unique exercise movements and respiratory techniques inherent to Baduanjin facilitate genuine physical and mental relaxation in practitioners. Furthermore, Baduanjin is frequently practiced in community-based group settings, which creates avenues for social interaction among participants. Sustained communication within such groups can stabilize and soothe the mental states of older adults, thereby contributing to the alleviation of negative psychological symptoms induced by their underlying illnesses.

*“I suffer from multiple chronic diseases. I used to feel nervous, worried, or panicked inexplicably. Later, my neighbors encouraged me to go to the community square to practice Baduanjin. Before and after practicing Baduanjin, we talk about both the things that make us happy and those that make us angry. Moreover, when practicing, I inhale and exhale deeply and slowly, synchronizing my breath with the rise and fall of the movements as well as the tensing and relaxing of the muscles. As a result, my entire body can relax, and the inner anxiety gradually fades away.” (P8).*

*“I am a gastric cancer patient and have been troubled by the condition since the operation. The gentle and smooth movements, combined with calm and slow breathing, make me forget my illness and relieve the heavy burden on my heart when I practice Baduanjin.” (P12).*

*“Especially in the practice of the seventh section, when exhaling slowly, punch slowly but forcefully while gradually widening my eyes. Visualize releasing my negative emotions through my eyes, breath, and fist. This made me feel extremely relaxed.” (P15).*

### Theme IV Enhancement of social functioning

#### Increased sharing and communication.

Participants of Baduanjin practice engaged in in-depth discussions regarding the core essentials of movements, respiratory techniques, and fitness-related efficacy, while also exchanging their practical experience. For instance, experienced practitioners may instruct novices on the appropriate regulation of respiration and the synchronization of movements with breathing rhythms to optimize training outcomes. Such interactive exchanges not only facilitate the improvement of novices’ practice proficiency but also strengthen mutual comprehension and trust during the interactive process.

*“Before and after the practice, we exchanged ideas on key points, feelings, and TCM theories. Additionally, we shared daily healthcare knowledge and discussed social and family issues.” (P9).*

#### Facilitation of conflict resolution.

Practitioners of Baduanjin integrate their lived experiences to share coping strategies and interpersonal skills. Through multidimensional approaches such as encouragement, guidance, consolation, and empathy, they provide advice from diverse perspectives, thereby facilitating mutual resolution of life-related conflicts.

*“My husband and I often quarrel in life. Practitioners advised me not to interfere too much in his life, not to impose my own opinions on him, but only to express my views and give corresponding advice. After that, I tried to follow their advice. As a result, there are fewer arguments between us, and we live in greater harmony.” (P4).*

#### Reduction of social isolation and loneliness.

Baduanjin is frequently practiced in a group context. Within such group-based exercise sessions, older adults engage in mutual communication, exchange experiential insights, expand their social networks, and strengthen interpersonal interactions. These processes enable them to acquire emotional support and a sense of belonging, thereby effectively mitigating social isolation and loneliness.

*“Baduanjin is a form of fitness qigong that inherits the essence of TCM culture. Through practicing Baduanjin, I can deeply experience the profoundness of Chinese culture and the wisdom of the ancients. It is like an old friend who has always accompanied me.” (P5).*

*“My husband died six months ago. I made a lot of friends by practicing Baduanjin, which made me no longer feel lonely.” (P20).*

### Theme V Appreciation of traditional culture

#### Recognition of traditional health-preserving culture.

Baduanjin, characterized by its rounded, dynamic-static integrated, and hardness-softness balanced movements, as well as its core guiding philosophy of simultaneous cultivation of physical form and spiritual essence along with harmony between humans and nature, centrally embodies the essence of traditional Chinese culture. This has thus attracted OAwCD in the community to persist in practicing it.

*“I am drawn to Baduanjin because its movements embody both the softness characterized by smoothness and fluidity, and the power marked by strength and steadiness, thereby presenting the characteristic of integrating rigidity and flexibility. The concept of equilibrium inherent in this form of exercise aligns with the ideological essence of ‘taking the middle path by avoiding extremes’ within the traditional Chinese doctrine of the Mean.” (P22).*

### Connection with traditional chinese medicine culture

Baduanjin is favored by a large number of older adults, primarily attributed to its embodiment of the profound traditional Chinese medical culture. Each movement of Baduanjin incorporates the Zang-Fu theory, meridian theory, and Qi-blood theory of TCM. It is not merely a simple form of physical exercise, but rather a vivid manifestation of a cultural symbol. For the elderly, practicing Baduanjin can not only nourish both the body and mind but also contribute to the inheritance of this precious cultural heritage.

*“I especially like the culture of TCM. I usually practice Baduanjin first and then Tai Chi. Both Baduanjin and Tai Chi are forms of fitness Qigong, and they complement each other. They make my life more fulfilling and interesting.” (P1).*

*“While practicing Baduanjin, I can personally experience the profound impact of TCM culture on human health. After each practice, I have different experiences. I can feel the flow of qi and blood, sense the rich TCM culture, and perceive the harmony and unity between man and nature.” (P21).*

## Discussion

Baduanjin is a traditional Chinese health-preserving qigong characterized by ease of learning and low-to-moderate exercise intensity, which is particularly suitable for community-dwelling OAwCD [41–42]. For older adults, low-to-moderate-intensity power training yields muscle function improvements comparable to those of high-intensity power training [43]. Notably, single bouts of exercise exceeding a certain intensity and duration can transiently increase reactive oxygen species (ROS) production from mitochondria and other oxidases, potentially inducing cellular damage [44], whereas chronic exercise mitigates age-related oxidative stress in multiple organs, including skeletal muscle [45]. This physiological basis supports the suitability of Baduanjin as a long-term exercise modality for OAwCD, and the present qualitative study explores the intrinsic determinants of their sustained practice from five core themes derived from participant interviews.

### Perceived Safety and Learning-Practice Ease: Core Determinants of Initial Adoption and Adherence

Perceived safety is a primary prerequisite for OAwCD to engage in and persist with any exercise program. This study reveals that persisting in Baduanjin practice has no side effects or additional physical burden on older adults, in line with previous studies [42,46–48]. For example, a participant with a history of knee joint replacement noted that Baduanjin did not cause joint injury and facilitated post-surgical recovery (P9), while another highlighted its safety advantage over more complex exercises (e.g., the Five-Animal Exercise) due to its simple movements and low fall risk (P2).

In addition, ease of learning and practice further reinforces adherence among OAwCD. Baduanjin’s slow-paced aerobic movements allow sufficient time for older adults to comprehend and execute each action, avoiding frustration associated with complex or fast-paced movements (P17). Its simplicity enables even older adults without prior exercise experience to quickly master basic movements (P9), and its flexibility in venue and timing—unrestricted by weather or expensive equipment—makes it accessible across diverse economic conditions and living environments (P5). Collectively, the perceived safety (low-to-moderate intensity, slow pace, no side effects) and ease of practice (simplified movements, flexible practice) eliminate key exercise barriers, serving as the primary drivers for OAwCD to adopt and sustain Baduanjin practice.

### Improvements in Physical Health: A Key Motivator for Sustained Practice

Interview findings confirm that Baduanjin exerts multifaceted positive effects on the physical health of OAwCD, directly reinforcing long-term adherence. Primarily, it enhances ROM: a participant with a history of cerebral infarction reported improved finger extension and lower limb ROM after one year of practice (P3), while another with cervical spondylosis and shoulder periarthritis noted reduced pain and increased arm elevation (P14). Additionally, Baduanjin enhances balance and coordination, with one participant reporting increased physical strength, flexibility, and reduced fear of falling (P13)—a critical benefit given the high fall risk among OAwCD.

Furthermore, it alleviates a range of somatic symptoms associated with chronic diseases. A participant with hyperlipidemia and constipation reported normalized bowel movements after twice-daily practice (P2), while others with conditions such as breast cancer, COPD, and anemia noted improved appetite, respiratory function, and peripheral circulation, respectively (P6, P11, P18). These physiological benefits directly address the core health concerns of OAwCD, forming a strong motivational cycle for sustained practice.

### Promotion of mental well-being: Mitigating psychological burdens of chronic illness

Baduanjin also plays a pivotal role in promoting mental well-being among OAwCD, which is often overlooked in chronic disease management. Its stretching and rounded movements alleviate somatic tension, while its deep, slow, uniform, and tranquil breathing pattern modulates the autonomic nervous system and stabilizes heart rate. Participants reported reduced anxiety and irritability through movement-breath coordination, noting a sense of relaxation and calmness after practice (P8, P19).

The requirement to focus on movement-respiration coordination and eliminate distracting thoughts further contributes to improved mood and reduced negative psychological states. A participant practicing in a quiet setting reported enhanced concentration and inner tranquility (P25), while a gastric cancer survivor noted that the gentle movements and calm breathing helped alleviate psychological burden and distract from illness-related distress (P12). Additionally, targeted movements (e.g., the seventh section) were reported to facilitate emotional release, further enhancing psychological well-being (P15). Notably, proactive learning of TCM theories embedded in Baduanjin was associated with improved cognitive function maintenance, as participants reported enhanced concentration, comprehension, and memory through focused practice (P17, P24).

### Enhancement of social functioning: Addressing isolation and loneliness

Social isolation and loneliness are prevalent concerns among older adults, particularly those with chronic diseases, as advancing age often leads to diminished social networks [13,49]. Group exercise programs have been shown to enhance social interaction and exercise adherence in this population [50–51], and Baduanjin is frequently practiced in group settings among OAwCD [41]. These groups, united by a shared interest in health promotion, provide a natural foundation for communication and mutual support.

Participants reported regular interactions before and after practice, encompassing not only exercise-related discussions but also sharing of health knowledge, life experiences, and emotional support (P9). For example, one participant noted that advice from peers improved interpersonal harmony with her spouse (P4). More importantly, group practice effectively mitigated social isolation and loneliness: a widowed participant reported forming new friendships through Baduanjin, eliminating feelings of loneliness (P20). The shared cultural and health-oriented values of Baduanjin further strengthen social bonds, fostering a sense of belonging and social participation (P5). Additionally, the mutual supervision inherent in group settings enhances long-term practice adherence, reinforcing the dual benefits of physical and social well-being.

### Appreciation of traditional culture: A unique emotional and motivational factor

Beyond its physical and psychological benefits, the cultural underpinnings of Baduanjin, which are grounded in TCM and traditional Chinese philosophy, represent a distinctive incentive for long-term adherence. As a key carrier of traditional health cultivation culture [20], Baduanjin is more than a set of physical movements; it is a cultural symbol that enables practitioners to engage with ancient TCM wisdom and perceive the harmony between the human body and nature (P21). Of the 25 participants, 9 expressed a positive attitude toward the TCM cultural elements embedded in Baduanjin.

The alignment of Baduanjin’s movement principles (integrating rigidity and flexibility) with traditional Chinese philosophical concepts (e.g., the Doctrine of the Mean) further deepens cultural identification (P22). For OAwCD, practicing Baduanjin enables active participation in cultural inheritance, satisfying not only physical exercise needs but also spiritual needs for cultural identity. This deep cultural connection fosters a psychological sense of belonging, complementing physical benefits to support long-term practice habits (P1).

## Strengths and limitations

This study provides in-depth qualitative insights into the perspectives of OAwCD regarding Baduanjin practice, systematically identifying five core themes underlying long-term adherence. This fills a research gap, as existing literature has predominantly focused on quantitative physical outcomes rather than the subjective determinants of practice persistence. Moreover, it highlights the holistic benefits of Baduanjin in addressing the multidimensional needs of OAwCD (physical, mental, social, and cultural), distinguishing it from single-focus exercises (e.g., simple stretching) that cannot meet the complex needs of this population. Finally, this study underscores the unique role of cultural connection in supporting exercise adherence among older adults, a factor often neglected in conventional health promotion strategies.

These findings carry important practical implications for promoting Baduanjin among community-dwelling older adults: (1) Emphasize safety, ease of learning and practice, and effectiveness to overcome barriers such as fear of injury and complex movements, through free community introductory sessions and simplified guidelines; (2) Organize community group practice to enhance social interaction and mutual support, thereby improving adherence; (3) Incorporate brief explanations of TCM concepts (e.g., meridian theory) to strengthen cultural identification; (4) Highlight holistic physical, mental, and social benefits to align with the multidimensional needs of older adults.

Several limitations of the present study should be acknowledged. First, all data were collected in Xinxiang, China, which restricts the generalizability of insights into factors and issues pertinent to a limited cohort of Baduanjin practitioners. Second, while data saturation was deemed achieved, given the inherent nature of qualitative research, the study may not encompass all potential scenarios. Accordingly, future research should undertake more comprehensive integration of quantitative and qualitative approaches, which will facilitate a more in-depth understanding of the factors underlying the persistence of Baduanjin practice among OAwCD. Additionally, although analyses were conducted in Chinese (rather than English), translations were verified by five researchers proficient in both languages; thus, the findings were not compromised by this aspect.

## Conclusion

The factors underlying the sustained practice of Baduanjin among community-dwelling OAwCD are multifaceted. Specifically, perceived safety, ease of learning and practice, physical health improvements (including enhanced range of motion, balance, coordination, and reduced somatic symptoms), promoted mental well-being, improved social functioning, and the intrinsic value embedded in traditional cultural heritage collectively serve as key motivators for their persistent engagement. These findings provide empirical support for the further promotion and implementation of‌‌ community-based Baduanjin exercise programs.

## Supporting information

S1 Appendix The eight sections of Baduanjin. (DOCX)

S2 Appendix Semi-structured interview guide for exploring perspectives of community-dwelling older adults with chronic diseases on Baduanjin practice. (DOCX)
