# Supplementary material for: Perspectives of community-dwelling older adults with chronic diseases on Baduanjin practice: A qualitative study
Source: PLoS One. 2026 Jun 12;21(6):e0351557. doi: 10.1371/journal.pone.0351557 (PMC13262812; doi:10.1371/journal.pone.0351557)
Supplement: S1 Appendix — (DOCX) [file pone.0351557.s001.docx]

Appendix 1: The Eight Sections of Baduanjin

Baduanjin consists of eight consecutive sections (Figure 1). The specific procedures are as follows:

1. Lifting Hands to Regulate the Sanjiao​

(Sanjiao encompasses the organs in the chest cavity, abdominal cavity, and pelvic cavity.)​

1. Drawing the Bow to Shoot the Eagle (Alternating Left and Right)​
2. Single-Hand Lifting to Regulate the Spleen and Stomach​
3. Looking Backward to Alleviate Five Fatigues and Seven Injuries​

(Five Fatigues refer to the fatigue of the five internal organs: heart, liver, spleen, lungs, and kidneys; Seven Injuries refer to the harm caused to the human body by excessive joy, anger, worry, overthinking, sorrow, fear, and fright.)​

1. Shaking the Head and Swinging the Tail to Reduce Heart Fire​
2. Clasping the Feet with Both Hands to Strengthen the Kidneys and Waist​
3. Making a Fist and Glaring to Boost Vital Energy​
4. Shaking the Body to Eliminate Various Ailments


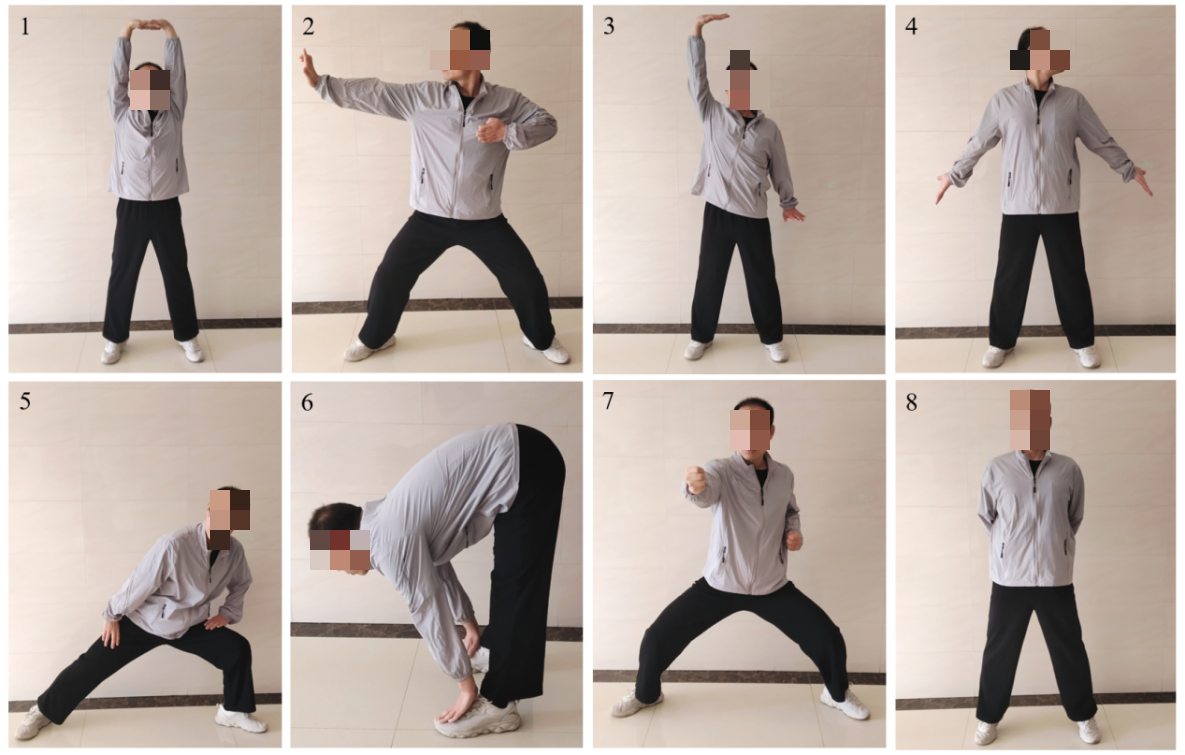


Figure 1: The Eight Sections of Baduanjin

(Note: The first author of this paper demonstrated these eight movements. )
