# Supplementary material for: Perspectives of community-dwelling older adults with chronic diseases on Baduanjin practice: A qualitative study
Source: PLoS One. 2026 Jun 12;21(6):e0351557. doi: 10.1371/journal.pone.0351557 (PMC13262812; doi:10.1371/journal.pone.0351557)
Supplement: S2 Appendix — (DOCX) [file pone.0351557.s002.docx]

Interview Guidelines for Perspectives of Community-dwelling Older Adults with Chronic Diseases on Baduanjin Practice

1. What are the reasons for practicing Baduanjin every day?

2. Can you tell me about your feelings and experiences in practicing Baduanjin?

3. What other factors do you think make you keep practicing Baduanjin?

4. What measures have you taken to make yourself better at practicing Baduanjin?
