# Supplementary material for: Perspectives of community-dwelling older adults with chronic diseases on Baduanjin practice: A qualitative study
Source: PLoS One. 2026 Jun 12;21(6):e0351557. doi: 10.1371/journal.pone.0351557 (PMC13262812; doi:10.1371/journal.pone.0351557)
Supplement: Attachment — Submitted filename: Response to Reviewers.docx [file pone.0351557.s003.docx]

Response to Academic Editors and Reviewers

Manuscript title: Perspectives of Community-dwelling Older Adults with Chronic Diseases on Baduanjin Practice: A Qualitative StudyManuscript ID: PONE-D-25-42202

Dear Editor and Reviewers,

Thank you very much for your constructive comments and valuable suggestions on our manuscript. We have carefully revised the manuscript according to all comments.

1. Response to Journal Requirements

1.1 PLOS ONE style requirements

We have carefully checked and revised the manuscript to fully comply with PLOS ONE’s formatting and style requirements, including file naming, structure, citations, and references, following the provided PLOS ONE templates.

1.2 Role of Funder

We have stated on lines 553–554 that the funders had no role in study design, data collection and analysis, decision to publish, or preparation of the manuscript.

1.3 Data availability (privacy issue)

The interview transcripts contain sensitive personal information of elderly participants, and public deposition would compromise participant privacy and breach the approval of our research ethics board. Therefore, the raw interview transcripts cannot be made publicly available. We have clearly stated this ethical restriction in the revised data availability statement.

1.4 Data sharing plan

We have revised the data availability statement to clarify that non-identifiable summarized data supporting the findings are included in the manuscript and supplementary materials. Restricted raw data are available from the corresponding author upon reasonable request and ethics committee approval, in compliance with PLOS ONE’s open data policy and ethical requirements.

1.5 Ethics statement

The ethics statement has been moved to and retained only in the Methods section, as required.

1.6 Supporting Information captions

We have added captions for all Supporting Information files at the end of the manuscript and updated corresponding in-text citations accordingly.

1.7 Citation of suggested references

We have reviewed the publications recommended by the reviewers and included those relevant to improve the context and contribution of this study.

2. Response to Reviewer #1

Comment 1: Expand the study purpose and academic contributions (line 82)

We have expanded the introduction to clearly state the specific academic contributions of this study beyond providing a foundation for future research:

• To reveal the lived experience and intrinsic motivation of community-dwelling older adults with chronic diseases practicing Baduanjin;

• To enrich the phenomenological understanding of traditional Chinese health-promoting exercises;

• To provide evidence for developing targeted interventions to promote healthy aging among older adults.

Comment 2: Add phenomenology as theoretical foundation

We have supplemented a detailed description of phenomenology in the Study design section, including its definition as a philosophy and theoretical lens, key characteristics relevant to this study, its application in qualitative research, and the rationale for using phenomenology as the theoretical foundation for this study. This description is consistent with Colaizzi's seven-step analytical framework.

Comment 3: Add detailed recruitment information (line 96)

We have added detailed information about participant recruitment, including how potential respondents were approached, cooperation with community managers as intermediaries, face-to-face invitations during routine Baduanjin practice, and the process of establishing trust and obtaining informed consent. These details have been integrated into the Methods section.

Comment 4: Add more verbatim responses

We have added additional representative participant verbatim quotations to strengthen the sections regarding “sharing and communication”, “facilitating conflict resolution”, and “appreciating traditional physical and health preserving culture”.

Comment 5: Rework the Discussion section

We have thoroughly revised the Discussion section as suggested:

• Content describing the benefits of Baduanjin (original lines 361–324 and 445–462) has been moved to the literature review before the methodology section;

• We have added in-depth discussion of the determinants of choosing Baduanjin, factors motivating sustained engagement, and unique characteristics of Baduanjin compared with other exercises;

• We have included discussion on how these determinants may foster greater participation and appreciation of Baduanjin among older adults in other communities.

Comment 6: Strengthen the Conclusion

We have rewritten and strengthened the Conclusion section to highlight the theoretical and practical contributions of this study to the scholarship, beyond simply reiterating the main themes. The revised conclusion reflects the nuance and depth of qualitative inquiry into the lived experience of older adults practicing Baduanjin.

3. Response to Reviewer #2

Comment 1: Use of AI in the Writing Process

We acknowledge that AI-assisted tools were used for language and grammatical corrections during the preparation of this manuscript. All authors confirm full responsibility for the content, logic, and conclusions of the manuscript.

Comment 2: Validation of themes (member checking)

We conducted a focused refinement of the extracted primary themes and their corresponding sub-themes to enhance their accuracy and academic rigor. Concurrently, we supplemented a detailed description of the theme validation procedures, including member checking and investigator triangulation, to ensure that the identified themes authentically and accurately reflect participants’ lived experiences.

Comment 3: Conceptualisation of themes / sub-themes

We have re-analyzed and refined all themes and subthemes to move beyond descriptive summaries toward phenomenological meaning units. They now capture essential and recurring patterns of lived experience as required in descriptive phenomenology.

Comment 4: Improve the Results section

We have further developed the Results section to clarify what each theme represents and how it reflects participants’ lived experiences. Narrative coherence within and across themes has been improved, with participant quotations used to illuminate and substantiate thematic meanings.

Comment 5: Improve synthesis for Theme 3

We have strengthened analytical synthesis for Theme 3, with clearer articulation of its core experiential meaning and better integration of quotations to support a coherent interpretation.

Comment 6: Data saturation and mixed-methods integration

• We have revised the conceptualization and description of data saturation to align with standard qualitative research practice.

• We have clarified the strengths of the study design and its contribution to knowledge in the strengths/limitations section.

We believe the manuscript has been significantly improved according to the editors’ and reviewers’ comments. We sincerely hope it is now suitable for publication in PLOS ONE.

Thank you again for your time and valuable feedback.

Sincerely,

Haixu Ji

Corresponding author

Email: [jihaixu@126.com](mailto:jihaixu@126.com)

Jinhua Zhang

Corresponding author

Email: [zjh.warm@163.com](mailto:zjh.warm@163.com)
