# Supplementary material for: Perspectives of community-dwelling older adults with chronic diseases on Baduanjin practice: A qualitative study
Source: PLoS One. 2026 Jun 12;21(6):e0351557. doi: 10.1371/journal.pone.0351557 (PMC13262812; doi:10.1371/journal.pone.0351557)
Supplement: Attachment — Submitted filename: Response_to_Reviewers_auresp_2.docx [file pone.0351557.s004.docx]

Response to Reviewer #2

Manuscript title: Perspectives of Community-dwelling Older Adults with Chronic Diseases on Baduanjin Practice: A Qualitative StudyManuscript ID: PONE-D-25-42202

Dear Reviewer,

Thank you very much for your professional, insightful comments and constructive suggestions. We fully agree with your methodological critique regarding the misalignment between our previous phenomenological claims and analytical outputs. We have carefully revised the manuscript following your valuable suggestions, with detailed revisions summarized as follows:

1. We have revised the methodology by adopting Braun and Clarke's thematic analysis, which better aligns with our actual analytical procedures and the established theme taxonomy. This revision makes the methodological framework epistemologically coherent and resolves the previous inconsistency between methodology and results.

2. We have retained the original five main themes and corresponding subthemes, as they adequately reflect participants’ perceptions, motivations, perceived benefits, and experiences of Baduanjin practice. All relevant descriptions throughout the manuscript have been revised to fit the logical framework of thematic analysis instead of phenomenological interpretation.

3. We have supplemented the descriptions of purposive sampling and data saturation in the Sampling and Recruitment section, and clarified that the sample size satisfies the standard requirements for qualitative thematic analysis.

Once again, we greatly appreciate your rigorous and insightful comments, which have substantially strengthened the methodological rigor and academic quality of our manuscript. The manuscript has been significantly improved in accordance with the suggestions provided by the editor and reviewers. We sincerely hope the revised version is now eligible for publication in *PLOS ONE*. Thank you once again for your time and valuable feedback.

Sincerely,

Haixu Ji

Corresponding author

Email: [jihaixu@126.com](mailto:jihaixu@126.com)

Jinhua Zhang

Corresponding author

Email: [zjh.warm@163.com](mailto:zjh.warm@163.com)
