# Supplementary material for: Perspectives of community-dwelling older adults with chronic diseases on Baduanjin practice: A qualitative study
Source: PLoS One. 2026 Jun 12;21(6):e0351557. doi: 10.1371/journal.pone.0351557 (PMC13262812; doi:10.1371/journal.pone.0351557)
Supplement: Peer review history

# Peer review: Decision Letter 0

**DOI:** 10.1371/journal.pone.0351557.r001

**Authors:** Assoc. Prof. Phakkharawat Sittiprapaporn

18 Feb 2026

PONE-D-25-42202Perspectives of Community-dwelling Older Adults with Chronic Diseases on Baduanjin Practice: A Qualitative StudyPLOS One

Dear Dr. Ji,

Thank you for submitting your manuscript to PLOS ONE. After careful consideration, we feel that it has merit but does not fully meet PLOS ONE’s publication criteria as it currently stands. Therefore, we invite you to submit a revised version of the manuscript that addresses the points raised during the review process.

Please submit your revised manuscript by Apr 04 2026 11:59PM. If you will need more time than this to complete your revisions, please reply to this message or contact the journal office at plosone@plos.org. When you're ready to submit your revision, log on to https://www.editorialmanager.com/pone/ and select the 'Submissions Needing Revision' folder to locate your manuscript file.

Please include the following items when submitting your revised manuscript:

- A letter that responds to each point raised by the academic editor and reviewer(s). You should upload this letter as a separate file labeled 'Response to Reviewers'.
- A marked-up copy of your manuscript that highlights changes made to the original version. You should upload this as a separate file labeled 'Revised Manuscript with Track Changes'.
- An unmarked version of your revised paper without tracked changes. You should upload this as a separate file labeled 'Manuscript'.

If you would like to make changes to your financial disclosure, please include your updated statement in your cover letter. Guidelines for resubmitting your figure files are available below the reviewer comments at the end of this letter.

If applicable, we recommend that you deposit your laboratory protocols in protocols.io to enhance the reproducibility of your results. Protocols.io assigns your protocol its own identifier (DOI) so that it can be cited independently in the future. For instructions see: https://journals.plos.org/plosone/s/submission-guidelines#loc-laboratory-protocols. Additionally, PLOS ONE offers an option for publishing peer-reviewed Lab Protocol articles, which describe protocols hosted on protocols.io. Read more information on sharing protocols at https://plos.org/protocols?utm_medium=editorial-email&utm_source=authorletters&utm_campaign=protocols.

We look forward to receiving your revised manuscript.

Kind regards,

Assoc. Prof. Phakkharawat Sittiprapaporn, Ph.D.

Academic Editor

PLOS One

Journal Requirements:

When submitting your revision, we need you to address these additional requirements.

1. Please ensure that your manuscript meets PLOS ONE's style requirements, including those for file naming. The PLOS ONE style templates can be found at

https://journals.plos.org/plosone/s/file?id=wjVg/PLOSOne_formatting_sample_main_body.pdf and

https://journals.plos.org/plosone/s/file?id=ba62/PLOSOne_formatting_sample_title_authors_affiliations.pdf

2. Thank you for stating the following financial disclosure: [This paper is supported by the Science and Technology Department of Henan Province. The project title is "Research on the Development Track and Promoting Mechanism of Self-management Behavior in Older Adults with Declining Intrinsic Ability", and the project number is 242102310167].

Please state what role the funders took in the study.  If the funders had no role, please state: ""The funders had no role in study design, data collection and analysis, decision to publish, or preparation of the manuscript.""

If this statement is not correct you must amend it as needed.

Please include this amended Role of Funder statement in your cover letter; we will change the online submission form on your behalf.

3. In the online submission form, you indicated that [The interview transcripts are not available due to privacy issues, but are available from the corresponding author on reasonable request.].

All PLOS journals now require all data underlying the findings described in their manuscript to be freely available to other researchers, either 1. In a public repository, 2. Within the manuscript itself, or 3. Uploaded as supplementary information.

This policy applies to all data except where public deposition would breach compliance with the protocol approved by your research ethics board. If your data cannot be made publicly available for ethical or legal reasons (e.g., public availability would compromise patient privacy), please explain your reasons on resubmission and your exemption request will be escalated for approval.

4. When completing the data availability statement of the submission form, you indicated that you will make your data available on acceptance. We strongly recommend all authors decide on a data sharing plan before acceptance, as the process can be lengthy and hold up publication timelines. Please note that, though access restrictions are acceptable now, your entire data will need to be made freely accessible if your manuscript is accepted for publication. This policy applies to all data except where public deposition would breach compliance with the protocol approved by your research ethics board. If you are unable to adhere to our open data policy, please kindly revise your statement to explain your reasoning and we will seek the editor's input on an exemption. Please be assured that, once you have provided your new statement, the assessment of your exemption will not hold up the peer review process.

5. Your ethics statement should only appear in the Methods section of your manuscript. If your ethics statement is written in any section besides the Methods, please move it to the Methods section and delete it from any other section. Please ensure that your ethics statement is included in your manuscript, as the ethics statement entered into the online submission form will not be published alongside your manuscript.

6. Please include captions for your Supporting Information files at the end of your manuscript, and update any in-text citations to match accordingly. Please see our Supporting Information guidelines for more information: http://journals.plos.org/plosone/s/supporting-information.

7. If the reviewer comments include a recommendation to cite specific previously published works, please review and evaluate these publications to determine whether they are relevant and should be cited. There is no requirement to cite these works unless the editor has indicated otherwise.

Reviewers' comments:

Reviewer's Responses to Questions

**Comments to the Author**

1. Is the manuscript technically sound, and do the data support the conclusions?

The manuscript must describe a technically sound piece of scientific research with data that supports the conclusions. Experiments must have been conducted rigorously, with appropriate controls, replication, and sample sizes. The conclusions must be drawn appropriately based on the data presented.

Reviewer #1: Partly

Reviewer #2: Yes

**********

2. Has the statistical analysis been performed appropriately and rigorously?

Reviewer #1: N/A

Reviewer #2: N/A

**********

3. Have the authors made all data underlying the findings in their manuscript fully available?

The PLOS Data policy requires authors to make all data underlying the findings described in their manuscript fully available without restriction, with rare exception (please refer to the Data Availability Statement in the manuscript PDF file). The data should be provided as part of the manuscript or its supporting information, or deposited to a public repository. For example, in addition to summary statistics, the data points behind means, medians and variance measures should be available. If there are restrictions on publicly sharing data—e.g. participant privacy or use of data from a third party—those must be specified.

Reviewer #1: Yes

Reviewer #2: Yes

**********

4. Is the manuscript presented in an intelligible fashion and written in standard English?

PLOS ONE does not copyedit accepted manuscripts, so the language in submitted articles must be clear, correct, and unambiguous. Any typographical or grammatical errors should be corrected at revision, so please note any specific errors here.

Reviewer #1: Yes

Reviewer #2: Yes

**********

5. Review Comments to the Author

Please use the space provided to explain your answers to the questions above. You may also include additional comments for the author, including concerns about dual publication, research ethics, or publication ethics. (Please upload your review as an attachment if it exceeds 20,000 characters)

Reviewer #1: Dear Authors,

Thank you for sharing your work.

SUGGESTIONS

1. In line 82 you have clearly stated the lack of exploration of this form of exercise as the purpose of this study. May I encourage you to expand on this please? In qualitative research a lack of exploration needs to be bolstered by a deeper argument. I would like to suggest to the authors they consider listing 2-3 contributions to academia that this exploration would bring aside from providing a foundation for future research. Thank you.

2. To strengthen the article, may I suggest the authors add in the Introduction section details regarding phenomenology? This would include a definition of phenomenology both as a philosophy and a theoretical lens, the characteristics of phenomenology as they pertain to the study, how phenomenology is utilized in qualitative research and how it is the most effective foundation for this study. I understand the authors are using Colaizzi's seven-step approach as the analytical framework and that's fine, but to align this study with phenomenology the authors should seriously consider expanding upon phenomenology as a theoretical foundation for this article. Thank you.

3. Line 96. The authors state the respondents were recruited from two communities in Xinxiang. This is good as it shows the authors' commitment to academia. However, there is a lack of detail of how the respondents were enrolled. Could the authors please add information regarding how the potential respondents were approached, were different types of media used, were heads of groups of organizations catering to the potential respondents approached to act as intermediaries to introduce the research, or did the authors approach the potential respondents individually when they practiced Baduanjin and managed to gain their confidence to enroll in the research? These details would uplift the methods section of the article. Thank you.

4. The verbatim responses of the participants is excellent. They add depth and nuance to the article. My suggestion is the section on "sharing and communication" could be strengthened by adding a couple more verbatim responses from the participants. The same for facilitating conflict resolution and appreciating the traditional physical and health preserving culture. Thank you.

5. The Discussion section needs to be reworked. May I make the following suggestions?

- A lot of the information in the sections from lines 361 to 324 and 445 to 462 expound on the benefits of Baduanjin. This is fine, but I think misplaced. Would the authors please consider reframing these lines as a literature review before the methodology section of the article? It would fit better there.

- May I request the authors discuss the findings of their research in the discussion section? Some pointers I would like to offer include (a) discuss the implications of the determinants of choosing to engage in Baduanjin as per the focus of the research stated by the authors, (b) discuss the findings and how these determinants continue to motivate the respondents to engage in Baduanjin, and (c) when compared to other forms of exercise how do the characteristics of Baduanjin motivate the respondents to keep engaging with this form of exercise?

- Please conjecture as to how these determinants may be used to foster better use and appreciation of Baduanjin for other older adults in other communities.

6. The conclusion of a qualitative article should do more than reiterate the main themes discovered. May I please encourage the authors to offer an insight into how this study of Baduanjin in Xinxiang among older adults contributes to scholarship aside from offering empirical support? Qualitative research is deeply nuanced, therefore, the conclusion of a qualitative article should reflect this nuance and depth of texture. May I request the authors please rewrite and strengthen this section. Thank you.

Reviewer #2: Reporting considerations

Use of AI in the Writing Process

There appear to be indicators that AI-assisted tools may have been used in the preparation of the manuscript. If this is the case, the authors should explicitly acknowledge and cite the use of AI, clarifying the nature and extent of its contribution.

Validation of themes

The manuscript does not clearly describe how the identified themes were validated with participants. It is important to explain how participants were engaged in member checking or other validation strategies to ensure that the themes accurately reflect their lived experiences.

Conceptualisation of themes / sub-themes

The themes and subthemes currently read more as topic summaries rather than phenomenological meaning units. In descriptive phenomenology, themes should capture essential and recurring patterns of meaning across the data, while subthemes should provide deeper, nuanced articulations of those meanings. The authors may wish to revisit the analysis to ensure that themes move beyond descriptive categorisation toward phenomenological essence.

Results

The results section would benefit from further development in articulating what each theme represents and how it captures participants’ lived experiences. Greater narrative coherence within and across themes would help convey a clearer analytic “story,” with participant quotations used to illuminate and substantiate the meanings of the themes rather than stand alone.

Theme 3...

Theme 3 relies heavily on extensive quotations, with limited analytical synthesis. While participant voice is essential, the theme would benefit from clearer articulation of what the theme represents and how the quotations collectively express a coherent experiential meaning.

Discussion

Use of Data Saturation

The discussion refers to data saturation in a manner that is not typically aligned with qualitative approaches. The authors may wish to reconsider how saturation is conceptualised and discussed, or clarify how it was operationalised within their methodological framework.

Integration of mixed methods (Strenghts / limitations)

Minor point - it is not clearly articulated how the mixed-methods design would enhance knowledge.

**********

6. PLOS authors have the option to publish the peer review history of their article (what does this mean?). If published, this will include your full peer review and any attached files.

If you choose “no”, your identity will remain anonymous but your review may still be made public.

**Do you want your identity to be public for this peer review?** For information about this choice, including consent withdrawal, please see our Privacy Policy.

Reviewer #1: No

Reviewer #2: **Yes:** Helen Ginman-Love

**********

[NOTE: If reviewer comments were submitted as an attachment file, they will be attached to this email and accessible via the submission site. Please log into your account, locate the manuscript record, and check for the action link "View Attachments". If this link does not appear, there are no attachment files.]

To ensure your figures meet our technical requirements, please review our figure guidelines: https://journals.plos.org/plosone/s/figures

You may also use PLOS’s free figure tool, NAAS, to help you prepare publication quality figures: https://journals.plos.org/plosone/s/figures#loc-tools-for-figure-preparation.

NAAS will assess whether your figures meet our technical requirements by comparing each figure against our figure specifications.

# Author comment: Author response to Decision Letter 1

**DOI:** 10.1371/journal.pone.0351557.r002

7 Apr 2026

Thank you very much for your constructive comments and valuable suggestions on our manuscript. We have carefully revised the manuscript according to all comments.

Attachment Submitted filename: Response to Reviewers.docx

# Peer review: Decision Letter 1

**DOI:** 10.1371/journal.pone.0351557.r003

**Authors:** Assoc. Prof. Phakkharawat Sittiprapaporn

11 May 2026

PONE-D-25-42202R1Perspectives of Community-dwelling Older Adults with Chronic Diseases on Baduanjin Practice: A Qualitative StudyPLOS One

Dear Dr. Ji,

Thank you for submitting your manuscript to PLOS ONE. After careful consideration, we feel that it has merit but does not fully meet PLOS ONE’s publication criteria as it currently stands. Therefore, we invite you to submit a revised version of the manuscript that addresses the points raised during the review process.

Please submit your revised manuscript by Jun 25 2026 11:59PM. If you will need more time than this to complete your revisions, please reply to this message or contact the journal office at plosone@plos.org. When you're ready to submit your revision, log on to https://www.editorialmanager.com/pone/ and select the 'Submissions Needing Revision' folder to locate your manuscript file.

Please include the following items when submitting your revised manuscript:

- A letter that responds to each point raised by the academic editor and reviewer(s). You should upload this letter as a separate file labeled 'Response to Reviewers'.
- A marked-up copy of your manuscript that highlights changes made to the original version. You should upload this as a separate file labeled 'Revised Manuscript with Track Changes'.
- An unmarked version of your revised paper without tracked changes. You should upload this as a separate file labeled 'Manuscript'.

If you would like to make changes to your financial disclosure, please include your updated statement in your cover letter. Guidelines for resubmitting your figure files are available below the reviewer comments at the end of this letter.

If applicable, we recommend that you deposit your laboratory protocols in protocols.io to enhance the reproducibility of your results. Protocols.io assigns your protocol its own identifier (DOI) so that it can be cited independently in the future. For instructions see: https://journals.plos.org/plosone/s/submission-guidelines#loc-laboratory-protocols. Additionally, PLOS ONE offers an option for publishing peer-reviewed Lab Protocol articles, which describe protocols hosted on protocols.io. Read more information on sharing protocols at https://plos.org/protocols?utm_medium=editorial-email&utm_source=authorletters&utm_campaign=protocols.

As the corresponding author, your ORCID iD is verified in the submission system and will appear in the published article. PLOS supports the use of ORCID, and we encourage all coauthors to register for an ORCID iD and use it as well. Please encourage your coauthors to verify their ORCID iD within the submission system before final acceptance, as unverified ORCID iDs will not appear in the published article. *Only* the individual author can complete the verification step; PLOS staff *cannot* verify ORCID iDs on behalf of authors.

We look forward to receiving your revised manuscript.

Kind regards,

Assoc. Prof. Phakkharawat Sittiprapaporn, Ph.D.

Academic Editor

PLOS One

Journal Requirements:

If the reviewer comments include a recommendation to cite specific previously published works, please review and evaluate these publications to determine whether they are relevant and should be cited. There is no requirement to cite these works unless the editor has indicated otherwise.

Additional Editor Comments:

Reviewers' comments:

Reviewer's Responses to Questions

**Comments to the Author**

1. If the authors have adequately addressed your comments raised in a previous round of review and you feel that this manuscript is now acceptable for publication, you may indicate that here to bypass the “Comments to the Author” section, enter your conflict of interest statement in the “Confidential to Editor” section, and submit your "Accept" recommendation.

Reviewer #1: All comments have been addressed

Reviewer #2: (No Response)

**********

2. Is the manuscript technically sound, and do the data support the conclusions?

The manuscript must describe a technically sound piece of scientific research with data that supports the conclusions. Experiments must have been conducted rigorously, with appropriate controls, replication, and sample sizes. The conclusions must be drawn appropriately based on the data presented.

Reviewer #1: Yes

Reviewer #2: Partly

**********

3. Has the statistical analysis been performed appropriately and rigorously?

Reviewer #1: Yes

Reviewer #2: N/A

**********

4. Have the authors made all data underlying the findings in their manuscript fully available?

The PLOS Data policy requires authors to make all data underlying the findings described in their manuscript fully available without restriction, with rare exception (please refer to the Data Availability Statement in the manuscript PDF file). The data should be provided as part of the manuscript or its supporting information, or deposited to a public repository. For example, in addition to summary statistics, the data points behind means, medians and variance measures should be available. If there are restrictions on publicly sharing data—e.g. participant privacy or use of data from a third party—those must be specified.

Reviewer #1: Yes

Reviewer #2: Yes

**********

5. Is the manuscript presented in an intelligible fashion and written in standard English?

PLOS ONE does not copyedit accepted manuscripts, so the language in submitted articles must be clear, correct, and unambiguous. Any typographical or grammatical errors should be corrected at revision, so please note any specific errors here.

Reviewer #1: Yes

Reviewer #2: Yes

**********

6. Review Comments to the Author

Reviewer #1: Thank you for your revisions. As this is a corrected version of your original submission you have addressed the concerns raised in the initial review process. No further revisions are required.

Reviewer #2: It is acknowledged that the authors have made revision to the manuscript overall and these revisions absolutely strengthen the manuscript.

As a reviewed, there still remains a misalignment between the methodology and analytical output. The authors state that Colaizzi's seven-step phenomenological method was used to analyse the data. However, the five themes presented in the Results section do not reflect the outputs of a genuine phenomenological enquiry. Instead, they constitute a taxonomy of motivational factors and perceived benefits. These are explanatory and functional categories, not phenomenological meaning structures.

Colaizzi's method, rooted in descriptive phenomenology, requires the researcher to move progressively from significant statements and formulated meanings toward an exhaustive description and ultimately a fundamental structure that captures the essential nature of the lived experience under investigation. The central question should be something like: what is it essentially like to persist in Baduanjin practice as an older adult living with chronic illness? The themes as currently presented do not answer this question, they answer the quite different question of why do people keep practicing, which is a motivational or behavioural research question more suited to other frameworks.

Notably, the fundamental structure of the phenomenon, a required outcome of Colaizzi's method, is entirely absent from the manuscript.

Specific concerns

1. Several themes read as outcome variables rather than experiential essences. "Improvements in physical health" and "Enhancement of social functioning" in particular describe what Baduanjin does rather than what it is like to experience it. A phenomenological theme should capture the texture and meaning of lived experience, not catalogue functional benefits.

2. The Discussion reinforces this problem by engaging primarily with biomedical and behavioural literature to validate the themes, rather than offering phenomenological interpretation of participants' lived worlds. Phrases such as "these physiological benefits directly address the core health concerns of OAwCD" reflect a positivist framing that is epistemologically inconsistent with phenomenological methodology.

3. The authors' response to Reviewer 2 notes that themes have been refined "to move beyond descriptive summaries toward phenomenological meaning units." However, this is not evident in the revised manuscript. The themes remain descriptive and categorical in character.

What the authors could do to address the issue:

Option 1: Commit fully to phenomenological methodology. This would require returning to the data and conducting a genuine phenomenological analysis. The participant quotations already contain material with real phenomenological depth descriptions of qi flow, sensing harmony with nature, forgetting illness during practice, experiencing cultural belonging. These could support meaning units around, for example, restored embodied agency in the face of chronic illness or practice as cultural self-recognition. The analysis should culminate in an exhaustive description and a clearly articulated fundamental structure of the lived experience. The Discussion should then offer phenomenological interpretation rather than biomedical validation.

Option 2: Reframe the study as thematic analysis. If a full phenomenological reworking is not feasible, the authors should honestly reframe their methodology as thematic analysis, which is well-suited to generating the kind of motivational and experiential theme taxonomy they have produced. This would require removing phenomenological claims throughout, adjusting the theoretical framing in the Study Design section accordingly, and ensuring the Discussion reflects thematic rather than phenomenological analytical logic. This is not a lesser choice, it is simply a more honest description of what was done, and the study would be methodologically sound on its own terms.

The current manuscript falls between these two approaches and is therefore methodologically vulnerable. The richness of the data and the clinical relevance of the topic mean this is worth revising carefully rather than abandoning.

**********

7. PLOS authors have the option to publish the peer review history of their article (what does this mean?). If published, this will include your full peer review and any attached files.

If you choose “no”, your identity will remain anonymous but your review may still be made public.

**Do you want your identity to be public for this peer review?** For information about this choice, including consent withdrawal, please see our Privacy Policy.

Reviewer #1: No

Reviewer #2: **Yes:** Helen Ginman-Love

**********

[NOTE: If reviewer comments were submitted as an attachment file, they will be attached to this email and accessible via the submission site. Please log into your account, locate the manuscript record, and check for the action link "View Attachments". If this link does not appear, there are no attachment files.]

To ensure your figures meet our technical requirements, please review our figure guidelines: https://journals.plos.org/plosone/s/figures

You may also use PLOS’s free figure tool, NAAS, to help you prepare publication quality figures: https://journals.plos.org/plosone/s/figures#loc-tools-for-figure-preparation.

NAAS will assess whether your figures meet our technical requirements by comparing each figure against our figure specifications.

# Author comment: Author response to Decision Letter 2

**DOI:** 10.1371/journal.pone.0351557.r004

14 May 2026

Thank you very much for your professional, insightful comments and constructive suggestions. We fully agree with your methodological critique regarding the misalignment between our previous phenomenological claims and analytical outputs. We have carefully revised the manuscript following your valuable suggestions.

Attachment Submitted filename: Response_to_Reviewers_auresp_2.docx

# Peer review: Decision Letter 2

**DOI:** 10.1371/journal.pone.0351557.r005

**Authors:** Assoc. Prof. Phakkharawat Sittiprapaporn

28 May 2026

Perspectives of Community-dwelling Older Adults with Chronic Diseases on Baduanjin Practice: A Qualitative Study

PONE-D-25-42202R2

Dear Dr. Ji,

We’re pleased to inform you that your manuscript has been judged scientifically suitable for publication and will be formally accepted for publication once it meets all outstanding technical requirements.

Within one week, you’ll receive an e-mail detailing the required amendments. When these have been addressed, you’ll receive a formal acceptance letter and your manuscript will be scheduled for publication.

An invoice will be generated when your article is formally accepted. Please note, if your institution has a publishing partnership with PLOS and your article meets the relevant criteria, all or part of your publication costs will be covered. Please make sure your user information is up-to-date by logging into Editorial Manager at Editorial Manager® and clicking the ‘Update My Information' link at the top of the page. For questions related to billing, please contact billing support.

If your institution or institutions have a press office, please notify them about your upcoming paper to help maximize its impact. If they’ll be preparing press materials, please inform our press team as soon as possible -- no later than 48 hours after receiving the formal acceptance. Your manuscript will remain under strict press embargo until 2 pm Eastern Time on the date of publication. For more information, please contact onepress@plos.org.

Kind regards,

Assoc. Prof. Phakkharawat Sittiprapaporn, Ph.D.

Academic Editor

PLOS One

Additional Editor Comments (optional):

Reviewers' comments:

Reviewer's Responses to Questions

**Comments to the Author**

1. If the authors have adequately addressed your comments raised in a previous round of review and you feel that this manuscript is now acceptable for publication, you may indicate that here to bypass the “Comments to the Author” section, enter your conflict of interest statement in the “Confidential to Editor” section, and submit your "Accept" recommendation.

Reviewer #2: All comments have been addressed

**********

2. Is the manuscript technically sound, and do the data support the conclusions?

The manuscript must describe a technically sound piece of scientific research with data that supports the conclusions. Experiments must have been conducted rigorously, with appropriate controls, replication, and sample sizes. The conclusions must be drawn appropriately based on the data presented.

Reviewer #2: Yes

**********

3. Has the statistical analysis been performed appropriately and rigorously?

Reviewer #2: Yes

**********

4. Have the authors made all data underlying the findings in their manuscript fully available?

The PLOS Data policy requires authors to make all data underlying the findings described in their manuscript fully available without restriction, with rare exception (please refer to the Data Availability Statement in the manuscript PDF file). The data should be provided as part of the manuscript or its supporting information, or deposited to a public repository. For example, in addition to summary statistics, the data points behind means, medians and variance measures should be available. If there are restrictions on publicly sharing data—e.g. participant privacy or use of data from a third party—those must be specified.

Reviewer #2: Yes

**********

5. Is the manuscript presented in an intelligible fashion and written in standard English?

PLOS ONE does not copyedit accepted manuscripts, so the language in submitted articles must be clear, correct, and unambiguous. Any typographical or grammatical errors should be corrected at revision, so please note any specific errors here.

Reviewer #2: Yes

**********

6. Review Comments to the Author

Please use the space provided to explain your answers to the questions above. You may also include additional comments for the author, including concerns about dual publication, research ethics, or publication ethics. (Please upload your review as an attachment if it exceeds 20,000 characters)

Reviewer #2: The authors have addressed the previous comments well - and the research is much stronger following those revisions.

**********

7. PLOS authors have the option to publish the peer review history of their article (what does this mean?). If published, this will include your full peer review and any attached files.

If you choose “no”, your identity will remain anonymous but your review may still be made public.

**Do you want your identity to be public for this peer review?** For information about this choice, including consent withdrawal, please see our Privacy Policy.

Reviewer #2: No

**********

# Editor report: Acceptance letter

**DOI:** 10.1371/journal.pone.0351557.r006

**Authors:** Assoc. Prof. Phakkharawat Sittiprapaporn

PONE-D-25-42202R2

PLOS One

Dear Dr. Ji,

I'm pleased to inform you that your manuscript has been deemed suitable for publication in PLOS One. Congratulations! Your manuscript is now being handed over to our production team.

At this stage, our production department will prepare your paper for publication. This includes ensuring the following:

* All references, tables, and figures are properly cited

* All relevant supporting information is included in the manuscript submission,

* There are no issues that prevent the paper from being properly typeset

You will receive further instructions from the production team, including instructions on how to review your proof when it is ready. Please keep in mind that we are working through a large volume of accepted articles, so please give us a few days to review your paper and let you know the next and final steps.

Lastly, if your institution or institutions have a press office, please let them know about your upcoming paper now to help maximize its impact. If they'll be preparing press materials, please inform our press team within the next 48 hours. Your manuscript will remain under strict press embargo until 2 pm Eastern Time on the date of publication. For more information, please contact onepress@plos.org.

You will receive an invoice from PLOS for your publication fee after your manuscript has reached the completed accept phase. If you receive an email requesting payment before acceptance or for any other service, this may be a phishing scheme. Learn how to identify phishing emails and protect your accounts at https://explore.plos.org/phishing.

If we can help with anything else, please email us at customercare@plos.org.

Thank you for submitting your work to PLOS ONE and supporting open access.

Kind regards,

PLOS ONE Editorial Office Staff

on behalf of

Assoc. Prof. Dr. Assoc. Prof. Phakkharawat Sittiprapaporn

Academic Editor

PLOS One
